# Efficacy of endotoxin absorption therapy on sepsis by polymyxin B-attached fibers

**DOI:** 10.1186/cc10411

**Published:** 2011-10-27

**Authors:** K Atagi

**Affiliations:** 1 Department of Critical Care Medicine, Osaka City General Hospital, Osaka, Japan

## Introduction

Endotoxin plays a role in the development of Gram-negative bacterial sepsis. In Japan, polymyxin B-attached fibers (PMX-B) are used clinically as an endotoxin absorption therapy to neutralize the biological activity of lipid A, the immunomodulatory center of lipopolysaccharide (LPS) endotoxin. Because hemodynamic improvement is not seen in all cases, it cannot be assumed that this therapy will be effective against all cases of sepsis.

## Hypothesis

Endotoxin absorption therapy is effective against abdominal infection. Moreover, the mortality rate significantly improved in endotoxin-positive cases of abdominal infection.

## Methods

Between 1997 and April 2008, endotoxin absorption therapy was performed on 105 septic patients in the ICU of Hyogo College of Medicine and the Osaka City General Hospital. The 105 cases were divided into an abdominal infection group (*n *= 45) and a nonabdominal infection group (*n *= 60). Before and after therapy, the endotoxin level was measured in patients using the limulus amoebocyte lysate (LAL) and endotoxin activity assay (EAA) methods. Moreover, we measured blood pressure, cardiac index, and the administered dose of catecholamine. Using a retrospective analysis, we compared Sequential Organ Failure Assessment (SOFA) scores; the Risk, Injury, Failure, Loss, and End stage (RIFLE) criteria; and the 28-day survival rate between the two groups.

## Results

After the endotoxin absorption therapy, mean blood pressure increased significantly from 67.9 ± 11.4 to 86.4 ± 6.3 mmHg in the abdominal infection group, whereas there was no change in the nonabdominal infection group. After the therapy, the SOFA scores and RIFLE criteria improved in both groups, but they improved significantly in the abdominal infection group. Patients in the abdominal infection group, especially the endotoxin-positive cases, recovered earlier from shock and had a significantly higher rate of survival than the abdominal infection group.

## Conclusion

In endotoxin-positive patients with an abdominal infection, absorption therapy improved survival rate and cardiac and renal dysfunction due to sepsis or septic shock. However, further studies are required to verify the effectiveness of endotoxin absorption therapy.

